# Supplementary material for: Proteomic Analysis of the Responses of Candida albicans during Infection of Galleria mellonella Larvae
Source: J Fungi (Basel). 2019 Jan 11;5(1):7. doi: 10.3390/jof5010007 (PMC6463115; doi:10.3390/jof5010007)
Supplement: Supplementary file 1 [file jof-05-00007-s001.pdf]

Supplementary Materials

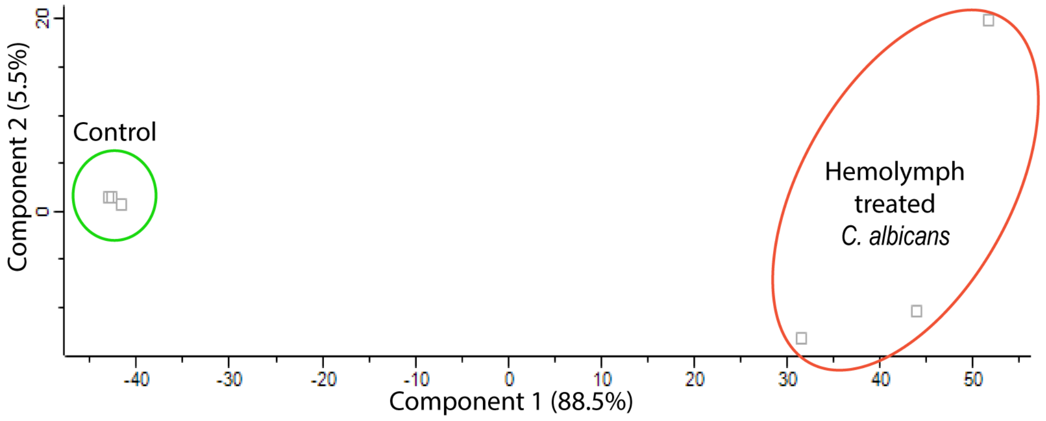

Figure S1: Shotgun quantitative proteomic analysis of *C. albicans* incubated in *G. mellonella* 100% hemolymph for 6 hours at 30 ^{o}C. Principal component analysis (PCA) of *C. albicans* incubated in 100% hemolymph and PBS for 6 hours with a clear distinction between control and treatment

Table S1: Functional enrichment of *C. albicans* proteins released during infection of *G. mellonella* larvae. *C. albicans* proteins found during in hemolymph during infection of *G. mellonella* larvae were grouped into functional categories based on the GO (Gene Ontology) annotations, using the FungiFun application.

Biological Processes

GO name	Exact p-value	Adjusted p-value	# genes / input
interaction with host	1.40E-08	6.8596e-7	6 / 101
cellular response to heat	0.011762	0.040686	3 / 101
tricarboxylic acid cycle	0.0111	0.039035	2 / 101
translational frameshifting	0.0090044	0.032202	1 / 101
intracellular steroid hormone receptor signaling pathway	0.0090044	0.032202	1 / 101
carbohydrate metabolic process	0.0063708	0.032202	4 / 101
cell wall organization	0.0061783	0.032202	3 / 101
filamentous growth of a population of unicellular organisms in response to neutral pH	0.0048813	0.032202	4 / 101
response to stress	0.004316	0.032202	3 / 101
pathogenesis	0.0036173	0.032202	9 / 101

Molecular Function

GO name	Exact p-value	Adjusted p-value	# genes / input
protein binding	1.9506e-8	6.8596e-7	6 / 101
voltage-gated anion channel activity	0.0090044	0.032202	1 / 101
phosphoglycerate kinase activity	0.0090044	0.032202	1 / 101
protein domain specific binding	0.0090044	0.032202	1 / 101
[acyl-carrier-protein] S-acetyltransferase activity	0.0090044	0.032202	1 / 101
[acyl-carrier-protein] S-malonyltransferase activity	0.0090044	0.032202	1 / 101
glucan endo-1,3-beta-D-glucosidase activity	0.0090044	0.032202	1 / 101
tripeptidase activity	0.0090044	0.032202	1 / 101
GTP binding	0.0063708	0.032202	4 / 101
nucleotide binding	0.0036272	0.032202	11 / 101

Cellular Component

GO name	Exact p-value	Adjusted p-value	# genes / input
cytoplasm	2.5692e-8	7.7444e-7	17 / 101
hyphal cell wall	1.0846e-10	7.6282e-9	10 / 101
fungal-type cell wall	7.0681e-12	7.4568e-10	12 / 101
extracellular region	1.9038e-8	6.8596e-7	11 / 101
cell surface	8.2643e-15	1.7438e-12	16 / 101

Table S2: List of proteins detected in *G. mellonella* larval hemolymph after 24 hours infection with *C. albicans* at 30 ^{o}C.

Uniprot ID	Protein Name	Score
Q59LF8	Uncharacterized protein	7.7409
A0A1D8PDX7	Uncharacterized protein	5.6692
A0A1D8PF42	Uncharacterized protein	5.9446
A0A1D8PST6	Uncharacterized protein	5.6758
Q59Q31	Uncharacterized protein	5.6966
Q59W37	Uncharacterized protein	5.6725
A0A1D8PGR8	Uncharacterized protein	7.9593
A0A1D8PL12	Uncharacterized protein	5.6592
A0A1D8PQF9	Uncharacterized protein	26.633
Q59WF2	Uncharacterized protein	5.6608
Q59WE9	Uncharacterized protein	6.1614
A0A1D8PMI5	Uncharacterized protein	5.6584
Q5A881	Uncharacterized protein	7.9593
Q5ANL8	Uncharacterized protein	5.6593
A0A1D8PI28	Uncharacterized protein	5.6614
A0A1D8PDN5	Uncharacterized protein	5.9529
A0A1D8PEH4	Uncharacterized protein	5.9325
A0A1D8PKC4	Ubiquitin-specific protease	6.8759
Q5A109	Ubiquitin-ribosomal 40S subunit protein S31 fusion protein	27.124
A0A1D8PC97	Tubulin beta chain	323.31
Q5ADR6	Translation initiation factor eIF2B subunit delta	5.6603
Q59P53	Translation factor GUF1, mitochondrial (EC 3.6.5.-) (Elongation factor 4 homolog) (EF-4) (GTPase GUF1) (Ribosomal back-translocase)	5.8389
A0A1D8PM35	Translation elongation factor 1 subunit beta	25.804
Q59N20	Transcription activator MSS11	6.1961
A0A1D8PJA8	Tos1p	164.21
A0A1D8PU69	Thioredoxin	44.721
Q5A4W7	Tetrafunctional fatty acid synthase subunit	5.9325
Q5A0X8	Surface antigen protein 2	39.745
Q5A2A1	Succinate dehydrogenase [ubiquinone] flavoprotein subunit, mitochondrial (EC 1.3.5.1)	192.87
Q59M48	Ste13p	5.8699
A0A1D8PSJ3	Sgd1p	7.9593
A0A1D8PQA0	Serine/threonine-protein phosphatase (EC 3.1.3.16)	6.2985
Q5AKV0	Serine C-palmitoyltransferase	5.7159
Q5AB48	Secreted protein RBT4 (PRY family protein 4) (Repressed by TUP1 protein 4)	260.77
Q59NP5	Secreted beta-glucosidase SUN41 (EC 3.2.1.-)	50.289
A0A1D8PQE5	RNA export factor	5.7826
A0A1D8PQ43	Ribonuclease H (RNase H) (EC 3.1.26.4)	6.2477
Q5ACL4	Restriction of telomere capping protein 1	5.9325
A0A1D8PTI2	Rab family GTPase	6.0995

P83775	Putative NADPH-dependent methylglyoxal reductase GRP2 (EC 1.1.1.283) (Cytoplasmic antigenic protein 2)	177.62
A0A1D8PJ20	Proteasome endopeptidase complex (EC 3.4.25.1)	5.7313
P46273	Phosphoglycerate kinase (EC 2.7.2.3)	8.9416
A0A1D8PRM7	Phosphoenolpyruvate carboxykinase	23.656
A0A1D8PEF9	Pfk26p	11.519
Q9Y7F0	Peroxiredoxin TSA1-A (EC 1.11.1.15) (Thiol-specific antioxidant protein) (Thioredoxin peroxidase)	7.4149
A0A1D8PS61	Opt3p	5.9325
Q59UZ4	Opt2p	5.9325
Q5AG68	Nucleoside diphosphate kinase (EC 2.7.4.6)	29.991
P83781	Mitochondrial outer membrane protein porin (Cytoplasmic antigenic protein 4)	6.7964
P83778	Malate dehydrogenase, cytoplasmic (EC 1.1.1.37)	40.507
A0A1D8PCJ7	Mak32p	5.6759
A0A1D8PS79	Isocitrate dehydrogenase [NADP] (EC 1.1.1.42)	12.734
P83777	Inorganic pyrophosphatase (EC 3.6.1.1) (Pyrophosphate phospho-hydrolase) (PPase)	7.022
A0A1D8PSZ0	Ife2p	5.9325
A0A1D8PG96	Hsp70 family ATPase	201.87
Q5A397	Hsp70 family ATPase	11.766
Q59VP1	Histone H2B.2	323.31
Q59VP2	Histone H2A.2	6.2119
A0A1D8PSA6	Histone deacetylase (EC 3.5.1.98)	5.6685
Q59VZ0	Hgt2p	8.0582
P46587	Heat shock protein SSA2	60.755
Q96VB9	Heat shock protein homolog SSE1 (Chaperone protein MSI3)	7.9593
P46598	Heat shock protein 90 homolog	55.408
Q59P43	GTP-binding nuclear protein	8.2306
Q5ADM7	Glyceraldehyde-3-phosphate dehydrogenase (EC 1.2.1.12)	55.376
Q9URB4	Fructose-bisphosphate aldolase (FBP aldolase) (FBPA) (EC 4.1.2.13) (37 kDa major allergen) (Fructose-1,6-bisphosphate aldolase) (IgE-binding allergen)	35.783
A0A1D8PT02	Flavodoxin-like fold family protein	74.887
P30575	Enolase 1 (EC 4.2.1.11) (2-phospho-D-glycerate hydro-lyase) (2-phosphoglycerate dehydratase)	100.19
Q5A0M4	Elongation factor 2 (EF-2)	30.184
Q59QD6	Elongation factor 1-alpha 2 (EF-1-alpha 2)	20.337
Q59UP3	Dur4p	5.9325
A0A1D8PME5	DNA-binding E3 ubiquitin-protein ligase	5.659
Q5AGX1	DNA repair protein	5.7247
A0A1D8PML0	DNA primase large subunit (EC 2.7.7.-)	5.7479
Q5AKA5	Cys-Gly metallodipeptidase DUG1 (EC 3.4.13.-) (Deficient in utilization of glutathione protein 1) (GSH degradosomal complex subunit DUG1)	6.7964
Q59M50	Cwt1p	5.7175
A0A1D8PSH3	Citrate synthase	6.126
A0A1D8PFK5	Chitin synthase	5.6786

Q5AF39	Cell wall protein PGA59 (GPI-anchored protein 59)	6.7964
Q5A1E0	Cell wall protein IFF5 (Adhesin-like protein IFF5)	6.7964
G1UB63	Cell wall protein 1 (Surface antigen protein 1) (Wall protein 1)	6.2214
Q59XX2	Cell surface mannoprotein MP65 (EC 3.2.1.-) (Mannoprotein of 65 kDa) (Soluble cell wall protein 10)	323.31
A0A1D8PM94	Ald6p	5.8108
A0A1D8PP43	Adh1p	6.6413
A0A1D8PFR4	Actin	323.31
Q59WG3	AAA family ATPase	31.856
A0A1D8PFG4	60S ribosomal protein L27	6.1451
A0A1D8PFS4	6-phosphogluconate dehydrogenase, decarboxylating (EC 1.1.1.44)	22.893
O42766	14-3-3 protein homolog	31.898

Table S3: Functional enrichment of proteins differentially abundant proteins from *C. albicans* exposed to *G. mellonella* larvae hemolymph. Proteins that were found statistically significant and differentially abundance in *C. albicans* exposed to 100% hemolymph were grouped into functional categories based on the GO (Gene Ontology) annotations, using the FungiFun application.

Biological process

GO name	Exact p-value	# genes / category
translation	6.6882e-35	58 / 128
formation of translation preinitiation complex	6.3724e-9	7 / 7
regulation of translational initiation	4.7997e-8	7 / 8
translational initiation	9.2525e-8	12 / 28
glycolytic process	1.8762e-7	10 / 20
protein folding	1.1493E-06	14 / 46
carbohydrate metabolic process	1.8874E-06	19 / 83
translational elongation	3.3663E-06	8 / 16
oxidation-reduction process	9.7092E-06	51 / 407
methionine biosynthetic process	0.000014358	7 / 14
GDP-mannose biosynthetic process	0.000021031	4 / 4
malate metabolic process	0.000021031	4 / 4
pyrimidine nucleotide biosynthetic process	0.000099493	4 / 5
tricarboxylic acid cycle	0.00010481	7 / 18
cellular amino acid biosynthetic process	0.00012384	10 / 37
interaction with host	0.00022661	7 / 20
sulfate assimilation	0.00031166	3 / 3
carbohydrate phosphorylation	0.0011525	5 / 13
fatty acid beta-oxidation	0.0011807	4 / 8
glutamine metabolic process	0.0011835	3 / 4
'de novo' pyrimidine nucleobase biosynthetic process	0.0011835	3 / 4
glyoxylate cycle	0.0011835	3 / 4
UDP-N-acetylglucosamine biosynthetic process	0.0011835	3 / 4
glycogen biosynthetic process	0.0011835	3 / 4
trehalose biosynthetic process	0.0011835	3 / 4
cellular carbohydrate metabolic process	0.0011835	3 / 4
cellular response to drug	0.0013596	37 / 328
L-methionine biosynthetic process from methylthioadenosine	0.0028096	3 / 5
cellular protein metabolic process	0.0031744	4 / 10
protein peptidyl-prolyl isomerization	0.0031744	4 / 10
phosphorylation	0.0034577	15 / 102
rRNA processing	0.0034921	11 / 64
ribosome biogenesis	0.0034921	11 / 64

induction by symbiont of host defense response	0.0044638	6 / 24
hydrogen sulfide biosynthetic process	0.0046075	2 / 2
mitochondrial electron transport, ubiquinol to cytochrome c	0.0046075	2 / 2
'de novo' UMP biosynthetic process	0.0046075	2 / 2
acetyl-CoA biosynthetic process	0.0046075	2 / 2
fructose 6-phosphate metabolic process	0.0046075	2 / 2
acetyl-CoA biosynthetic process from acetate	0.0046075	2 / 2
hydrogen ion transmembrane transport	0.0047234	4 / 11
response to toxic substance	0.0053367	3 / 6
regulation of actin filament polymerization	0.0053367	3 / 6

Molecular function

GO name	Exact p-value	Adjusted p-value	# genes / category
structural constituent of ribosome	1.5601e-21	3.2372e-19	36 / 81
nucleotide binding	3.8332e-9	3.977e-7	68 / 490
lyase activity	5.7546e-8	2.9852E-06	13 / 32
RNA binding	7.1461e-8	3.489E-06	30 / 152
translation initiation factor activity	9.2525e-8	4.0419E-06	12 / 28
catalytic activity	5.7322e-7	2.2656E-05	46 / 319
isomerase activity	6.1527E-06	0.00018238	11 / 33
translation elongation factor activity	6.1685E-05	0.0015515	6 / 12
transferase activity	6.4532E-05	0.0015753	36 / 271
ATP binding	0.00014516	0.0030893	53 / 473
L-malate dehydrogenase activity	0.00031166	0.0056234	3 / 3
malate dehydrogenase activity	0.00031166	0.0056234	3 / 3
unfolded protein binding	0.00039326	0.0068002	8 / 28
peptidyl-prolyl cis-trans isomerase activity	0.00046366	0.0078539	5 / 11
cytochrome-c oxidase activity	0.00062369	0.010353	4 / 7
metallopeptidase activity	0.00087226	0.013756	6 / 18
oxidoreductase activity	0.0008784	0.013756	36 / 309
metal ion binding	0.00092029	0.014145	45 / 414
kinase activity	0.00097425	0.014702	14 / 81
oxidoreductase activity, acting on the CH-OH group of donors, NAD or NADP as acceptor	0.0010464	0.014884	8 / 32
succinate dehydrogenase activity	0.0011835	0.014884	3 / 4
GTP binding	0.0012476	0.015456	14 / 83
protein binding	0.0021413	0.024684	6 / 21
ligase activity	0.0030085	0.033294	10 / 54
ATP-dependent helicase activity	0.0032905	0.034571	7 / 30
flavin adenine dinucleotide binding	0.0040265	0.037128	8 / 39
trehalose-phosphatase activity	0.0046075	0.037128	2 / 2
cytochrome-b5 reductase activity, acting on NAD(P)H	0.0046075	0.037128	2 / 2

AMP binding	0.0046075	0.037128	2 / 2
6-phosphofructokinase activity	0.0046075	0.037128	2 / 2
mannose-1-phosphate guanylyltransferase activity	0.0046075	0.037128	2 / 2
acetate-CoA ligase activity	0.0046075	0.037128	2 / 2
FK506 binding	0.0046075	0.037128	2 / 2
oxidoreductase activity, acting on a sulfur group of donors, NAD(P) as acceptor	0.0046075	0.037128	2 / 2
pyruvate dehydrogenase (acetyl-transferring) activity	0.0046075	0.037128	2 / 2
3-dehydroquinate dehydratase activity	0.0046075	0.037128	2 / 2
NAD binding	0.0048362	0.038229	7 / 32
peptidase activity	0.005024	0.039339	11 / 67
hydrolase activity	0.0053339	0.040268	35 / 331
peptide binding	0.0053367	0.040268	3 / 6
GTPase activity	0.0064613	0.048314	8 / 42
aminopeptidase activity	0.0067097	0.049724	4 / 12

Cellular Component

**GO name**	**Exact p-value**	**Adjusted p-value**	**# genes / category**
cytoplasm	3.3477e-39	2.7786e-36	106 / 393
ribosome	6.1521e-22	1.7021e-19	37 / 84
ribonucleoprotein complex	3.7798e-15	6.2745e-13	28 / 72
cell surface	1.1072e-11	1.5316e-9	33 / 129
eukaryotic translation initiation factor 3 complex	4.2598e-10	5.0509e-8	8 / 8
eukaryotic 43S preinitiation complex	6.3724e-9	4.8083e-7	7 / 7
eukaryotic 48S preinitiation complex	6.3724e-9	4.8083e-7	7 / 7
intracellular	9.5751e-9	6.6228e-7	29 / 132
yeast-form cell wall	1.0982e-8	7.0116e-7	16 / 44
cytosol	5.1594e-8	2.8549E-06	14 / 37
plasma membrane	9.0272e-7	3.4057E-05	46 / 324
hyphal cell wall	2.305E-06	7.6527E-05	16 / 62
mitochondrion	5.2393E-06	0.00016106	29 / 175
large ribosomal subunit	0.00014177	0.0030893	5 / 9
fungal-type cell wall	0.00017938	0.0037222	16 / 86
hyphal septin ring	0.00031166	0.0056234	3 / 3
cytosolic small ribosomal subunit	0.00031166	0.0056234	3 / 3
small-subunit processome	0.00039326	0.0068002	8 / 28
cytoskeleton	0.00083335	0.013562	8 / 31
peroxisome	0.001621	0.019499	6 / 20
chromosome	0.0016826	0.019951	9 / 42
nucleosome	0.0020118	0.023518	4 / 9
mitochondrial outer membrane	0.0024005	0.027294	5 / 15
small ribosomal subunit	0.0031744	0.033779	4 / 10

nucleus	0.0042815	0.037128	59 / 622
cellular bud neck septin structure	0.0046075	0.037128	2 / 2
6-phosphofructokinase complex	0.0046075	0.037128	2 / 2

Table S4: List of proteins detected which were statistically significant and differentially in *C. albicans* incubated in *G. mellonella* hemolymph as compared to PBS after 6 hours incubated at 30 ^{o}C

(A) Proteins increased in abundance in *C. albicans* incubated in *G. mellonella* hemolymph.

Fold Change (+)	Uniprot ID	Protein Name
44.4	A0A1D8PL15	D-arabinose 1-dehydrogenase
12.1	Q59VX7	Karyopherin beta
11.6	A0A1D8PEW7	Uncharacterized protein
8.3	Q5AND9	Arf family GTPase
8.0	A0A1D8PGR6	5-oxoprolinase
7.9	A0A1D8PLT4	Karyopherin
7.8	Q5AI87	Carboxymethylenebutenolidase
7.7	Q59M69	ATP-dependent
7.6	Q59K86	3-hydroxyanthranilate 3,4-dioxygenase
7.5	A0A1D8PIF8	Leu42p
7.4	Q59SI4	Nmd5p
7.2	A0A1D8PTB8	Copper metallochaperone
7.0	A0A1D8PR39	Ahp2p
6.8	A0A1D8PSW2	Threonine aldolase
5.9	A0A1D8PMF7	Uncharacterized protein
5.8	A0A1D8PC97	Tubulin beta chain
5.8	Q5ALW6	Glutamine-dependent NAD+) synthetase
5.7	A0A1D8PN39	Uncharacterized protein
5.6	A0A1D8PNJ2	Uncharacterized protein
5.2	A0A1D8PHY7	Uncharacterized protein
5.2	A0A1D8PGP5	Uncharacterized protein
4.9	A0A1D8PDB0	Proteasome regulatory particle lid subunit
4.9	A0A1D8PE84	Cdr4p
4.7	A0A1D8PJF0	Uncharacterized protein
4.7	Q5A015	Riboflavin kinase
4.6	A0A1D8PLD7	Uncharacterized protein
4.5	A0A1D8PU69	Thioredoxin
4.3	Q5A8A6	Carbamoyl-phosphate synthase Glutamine-hydrolyzing)
4.2	A0A1D8PE78	Importin-alpha export receptor
4.1	Q5ADN1	Protein phosphatase 2A structural subunit
4.0	A0A1D8PLE6	Uncharacterized protein
3.9	A0A1D8PI19	Phm7p
3.8	A0A1D8PH17	V-type proton ATPase subunit F
3.7	Q5AM80	Enolase-phosphatase E1
3.7	A0A1D8PRI0	Nicotinate phosphoribosyltransferase
3.7	Q5AG73	Methylthioribulose-1-phosphate dehydratase MTRu-1-P

		dehydratase
3.6	A0A1D8PPX6	Uncharacterized protein
3.6	Q59RH5	Histone acetyltransferase type B subunit 2
3.5	Q59PE7	Protein BCP1
3.4	Q92209	Homoserine kinase
3.4	A0A1D8PDA4	Fma1p
3.4	A0A1D8PNK0	Dap2p
3.3	A0A1D8PHR5	Pst1p
3.3	A0A1D8PHQ6	Gre2p
3.3	A0A1D8PTY0	Uncharacterized protein
3.3	Q59NN8	Hsp70 nucleotide exchange factor FES1
3.2	A0A1D8PTV7	Putative cystathionine beta-lyase
3.2	A0A1D8PQ94	Hsp90 cochaperone
3.2	Q5A5B2	Uncharacterized protein
3.1	A0A1D8PCY4	Ecm33p
3.1	Q5AH60	tRNA (guanine-N7)-)-methyltransferase non-catalytic subunit TRM82
3.1	Q5AMR6	Uncharacterized protein
3.0	A0A1D8PQD4	E2 ubiquitin-conjugating protein
3.0	P56553	Cell growth-regulated gene 1 protein
3.0	Q5AF44	Thioredoxin peroxidase
3.0	A0A1D8PDL7	Uncharacterized protein
3.0	A0A1D8PFI3	Ribokinase
2.9	A0A1D8PGI9	Ubiquitinyl hydrolase 1
2.9	A0A1D8PT84	Uncharacterized protein
2.9	Q59Z17	Catabolic 3-dehydroquinase (cDHQase)
2.8	Q5A786	Profilin
2.7	Q59Z50	Spermidine synthase
2.7	A0A1D8PFU8	Uncharacterized protein
2.7	A0A1D8PFV6	Uncharacterized protein
2.7	A0A1D8PPB1	Ardp
2.7	A0A1D8PE63	Glutamate--cysteine ligase
2.7	G1UAZ9	Uncharacterized protein
2.5	A0A1D8PRF3	Uncharacterized protein
2.5	A0A1D8PNZ4	Cup1p
2.5	P0CY19	Deoxyuridine 5'-triphosphate nucleotidohydrolase
2.5	Q5A362	Cystathionine gamma-lyase
2.5	A0A1D8PJD2	Deoxyhypusine synthase
2.5	P87219	Sorbose reductase SOU1
2.5	Q59US5	Bifunctional cysteine synthase/O-acetylhomoserine aminocarboxypropyltransferase
2.4	A0A1D8PLH0	Phosphomevalonate kinase
2.4	A0A1D8PJD7	GTPase-activating protein
2.4	Q59Z55	Uncharacterized protein
2.4	Q59S63	tRNA pseudouridine synthase 1

2.4	Q59U89	Uncharacterized protein
2.4	Q5AHF9	Glucosamine 6-phosphate N-acetyltransferase
2.4	Q59P52	Phosphoserine aminotransferase
2.4	P43075	tRNA ligase
2.4	Q5ACY8	4a-hydroxytetrahydrobiopterin dehydratase
2.4	P28870	FK506-binding protein 1
2.3	A0A1D8PHW0	Sedoheptulose-bisphosphatase
2.3	A0A1D8PE37	Amidophosphoribosyltransferase
2.3	Q5AK98	Nucleotidase
2.3	A0A1D8PFF9	E2 ubiquitin-conjugating protein
2.3	A0A1D8PFS4	6-phosphogluconate dehydrogenase, decarboxylating
2.3	A0A1D8PKJ3	E1 ubiquitin-activating protein
2.3	Q5ABB2	Lactoylglutathione lyase
2.3	Q5ALX8	Adenine phosphoribosyltransferase
2.3	Q59N80	Inosine triphosphate pyrophosphatase
2.3	Q5A0L4	Plc2p
2.2	A0A1D8PKW2	Fructose 1,6-bisphosphate 1-phosphatase
2.2	A0A1D8PP67	Riboflavin synthase
2.2	A0A1D8PH39	Uncharacterized protein
2.2	A0A1D8PLI2	Isopentenyl-diphosphate delta-isomerase
2.2	P46587	Heat shock protein SSA2
2.1	Q59X24	Exopolyphosphatase
2.1	Q5AA13	Gim5p
2.1	Q59R27	Uncharacterized protein
2.1	A0A1D8PPG2	Uncharacterized protein
2.1	A0A1D8PHE5	Uncharacterized protein
2.1	Q5AMP4	Malate dehydrogenase
2.1	Q59MN2	Bifunctional 4-alpha-glucanotransferase/amylo-alpha-1,6-glucosidase
2.1	Q59VY8	Galactokinase
2.1	A0A1D8PJK5	D-lactate dehydrogenase
2.1	Q59T95	Cystathionine beta-synthase
2.1	Q5A934	Zinc finger-containing protein
2.1	A0A1D8PCS7	Putative pyridoxal 5'-phosphate synthase
2.1	P31225	Corticosteroid-binding protein
2.1	A0A1D8PNS0	Guanylate kinase
2.1	A0A1D8PH42	Branched-chain-amino-acid aminotransferase
2.1	A0A1D8PSA9	Phosphoglucomutase
2.0	Q5ANE2	Uncharacterized protein
2.0	Q59WC5	4-nitrophenylphosphatase
2.0	A0A1D8PD11	Uncharacterized protein
2.0	Q5ABA2	Survival factor 1
2.0	A0A1D8PU04	S-formylglutathione hydrolase
2.0	Q5A1M1	Tfs1p
2.0	A0A1D8PKY7	Histidine biosynthesis trifunctional protein

2.0	Q59NB8	Leukotriene A-4 hydrolase homolog
2.0	P0CH96	Adenylosuccinate synthetase
2.0	A0A1D8PSE7	Ifr2p
2.0	Q5A8Z4	Superoxide dismutase
2.0	Q96VB9	Heat shock protein homolog SSE1
2.0	A0A1D8PH55	4-aminobutyrate transaminase
2.0	P0CY20	3'2'),5'-bisphosphate nucleotidase 1
2.0	P83778	Malate dehydrogenase, cytoplasmic
2.0	A0A1D8PT02	Flavodoxin-like fold family protein
2.0	A0A1D8PQB4	Dipeptidyl peptidase 3
2.0	A0A1D8PKV4	Fum12p
2.0	Q5APF2	GMP synthase [glutamine-hydrolyzing]
2.0	A0A1D8PPK1	Ebp1p
1.9	Q59MZ5	Phosphoribosylformylglycinamidine synthase
1.9	Q5AKA5	Cys-Gly metallodipeptidase DUG1
1.9	P46598	Heat shock protein 90 homolog
1.9	Q5AIA6	Pyridoxine biosynthesis protein
1.9	Q5ANE7	Pin3p
1.9	P22011	Peptidyl-prolyl cis-trans isomerase
1.9	A0A1D8PMP0	Oye32p
1.9	P43076	pH-responsive protein 1
1.9	A0A1D8PQ57	Bifunctional AP-4-A phosphorylase/ADP sulfurylase
1.9	A0A1D8PLC6	Putative methyltransferase
1.9	P41797	Heat shock protein SSA1
1.9	A0A1D8PFY5	Putative Xaa-Pro dipeptidase
1.9	P83780	Glucose-6-phosphate isomerase
1.9	A0A1D8PPR2	2-deoxyglucose-6-phosphatase
1.9	A0A1D8PIB2	Asparagine synthase
1.9	Q5ADN2	Putative phosphomutase
1.9	Q5A4M2	Malate dehydrogenase
1.9	A0A1D8PRQ4	Uncharacterized protein
1.8	P83775	Putative NADPH-dependent methylglyoxal reductase GRP2
1.8	A0A1D8PHE6	Uncharacterized protein
1.8	A0A1D8PQN3	Long-chain fatty acid transporter
1.8	A0A1D8PQH5	Superoxide dismutase
1.8	Q59SU1	Candidapepsin-9
1.8	A0A1D8PQI4	Ribulose-phosphate 3-epimerase
1.8	Q59T35	Osm1p
1.8	Q59T45	Putative amidotransferase
1.8	Q5AG68	Nucleoside diphosphate kinase
1.8	A0A1D8PTD8	Thiosulfate sulfurtransferase
1.8	A0A1D8PHU6	Putative phosphoric monoester hydrolase
1.8	A0A1D8PQE6	Uncharacterized protein
1.8	O42766	14-3-3 protein homolog
1.8	A0A1D8PCD2	Nma111p

1.8	A0A1D8PIF6	Sulfite reductase subunit alpha
1.8	A0A1D8PRJ1	Uncharacterized protein
1.8	Q5ALM6	Peptidyl-prolyl cis-trans isomerase
1.8	Q5A500	Adenylyl-sulfate kinase
1.8	A0A1D8PNG6	UDP-N-acetylglucosamine diphosphorylase
1.8	Q5AND4	Rdi1p
1.7	P30575	Enolase 1
1.7	Q5APD5	Uncharacterized protein
1.7	P82612	Phosphoglycerate mutase
1.7	Q59MR4	Coproporphyrinogen oxidase
1.7	P46273	Phosphoglycerate kinase
1.7	P82610	5-methyltetrahydropteroyltriglutamate--homocysteine methyltransferase
1.7	A0A1D8PGS7	Phosphoribosylglycinamide formyltransferase
1.7	Q9P975	Eukaryotic translation initiation factor 4E
1.7	A0A1D8PI24	D-arabinose 1-dehydrogenase
1.7	A0A1D8PL85	Aspartate aminotransferase
1.7	Q9URB4	Fructose-bisphosphate aldolase
1.7	A0A1D8PP00	Arginase
1.7	A0A1D8PGN7	Hypoxanthine phosphoribosyltransferase
1.7	Q59US8	Uncharacterized protein
1.7	Q5ABA5	Uncharacterized protein
1.7	Q5AK88	Uncharacterized protein
1.7	P83784	Heat shock protein SSC1, mitochondrial
1.7	Q5ABP8	Protein ROT1
1.7	P83776	Hexokinase-2
1.7	A0A1D8PKZ7	Pyrroline-5-carboxylate reductase
1.7	A0A1D8PGT5	Aldehyde dehydrogenase
1.7	Q5A784	Ofr1p
1.7	A0A1D8PMB3	Xylulokinase
1.7	Q59PZ6	6-phosphogluconolactonase
1.7	Q5A860	Translationally-controlled tumor protein homolog
1.7	O13318	pH-responsive protein 2
1.7	Q59KZ1	Aminopeptidase 2
1.7	A0A1D8PM15	Uncharacterized protein
1.6	P13649	Orotidine 5'-phosphate decarboxylase
1.6	Q59R31	Argininosuccinate lyase
1.6	A0A1D8PLY4	Pyruvate carboxylase
1.6	A0A1D8PEM3	Doa1p
1.6	Q5A2A7	Metalloendopeptidase
1.6	Q5AG89	Thioredoxin reductase
1.6	Q5A6L1	Fumarase
1.6	A0A1D8PF30	Tubulin-binding prefolding complex subunit
1.6	A0A1D8PPI6	Cip1p
1.6	P31353	Phosphomannomutase

1.6	Q5ADP5	ADP-ribose diphosphatase
1.6	Q5AKW4	Phosphoacetylglucosamine mutase
1.6	Q9Y7F0	Peroxiredoxin TSA1-A
1.6	A0A1D8PS11	Orotate phosphoribosyltransferase
1.6	Q5A750	Transketolase
1.6	A0A1D8PKJ4	Saccharopine dehydrogenase
1.6	Q5ADT4	Glycerol 2-dehydrogenase
1.6	A0A1D8PH00	Asr3p
1.6	A0A1D8PQK5	3-isopropylmalate dehydrogenase
1.6	A0A1D8PL14	Ornithine-oxo-acid transaminase
1.6	P34948	Mannose-6-phosphate isomerase
1.6	A0A1D8PCV7	L-aminoadipate-semialdehyde dehydrogenase
1.6	A0A1D8PPG7	Ketol-acid reductoisomerase, mitochondrial
1.6	Q59NQ5	Glutathione-disulfide reductase
1.6	Q5A7S3	Uncharacterized protein
1.6	Q59WG6	Aspartyl aminopeptidase
1.6	A0A1D8PQ26	Adenosine kinase
1.6	P83773	Acetyl-CoA hydrolase
1.6	P83783	Adenosylhomocysteinase
1.6	A0A1D8PNG9	Threonine synthase
1.6	A0A1D8PEI2	Dihydroxyacetone kinase
1.6	P83779	Pyruvate decarboxylase
1.6	A0A1D8PES3	Ran GTPase-binding protein
1.6	P83777	Inorganic pyrophosphatase
1.6	Q59Z14	Deoxyhypusine hydroxylase
1.6	A0A1D8PLJ3	Superoxide dismutase [Cu-Zn]
1.6	Q5A435	Prefoldin subunit 4
1.6	A0A1D8PCN0	Bifunctional UDP-glucose 4-epimerase/aldose 1-epimerase
1.6	Q5A1Q0	Glucose-6-phosphate 1-epimerase
1.5	A0A1D8PSU2	Pyridoxamine-phosphate oxidase
1.5	A0A1D8PMF8	Calmodulin
1.5	Q59TZ8	Phosphotransferase
1.5	A0A1D8PTR7	Tropomyosin
1.5	Q5A017	Transaldolase
1.5	A0A1D8PGS5	Isocitrate dehydrogenase [NAD] subunit, mitochondrial
1.5	Q59WG0	Adenosine 5'-monophosphoramidase
1.5	Q5A330	Pex19p
1.5	A0A1D8PNP4	Uncharacterized protein
1.5	A0A1D8PRM7	Phosphoenolpyruvate carboxykinase
1.5	A0A1D8PRR5	Argininosuccinate synthase
1.5	Q5AL34	Psa2p
1.5	A0A1D8PHC9	Aspartate transaminase
1.5	A0A1D8PFX8	Rab GDP dissociation inhibitor
1.5	P79023	Phospho-2-dehydro-3-deoxyheptonate aldolase, tyrosine-inhibited

1.5	A0A1D8PE67	Bifunctional aminoimidazole ribotide synthase/glycinamide ribotide synthase
1.5	A0A1D8PP43	Adh1p
1.5	Q59QN6	Formate dehydrogenase
1.5	A0A1D8PHS4	Uncharacterized protein
1.5	A0A1D8PSH3	Citrate synthase

(B) Proteins decreased in abundance in *C. albicans* incubated in *G. mellonella* hemolymph.

**Fold Change (-)**	**Uniprot ID**	**Protein Name**
374.0	A0A1D8PF90	Uncharacterized protein
259.6	Q5AB84	Uncharacterized protein
235.3	Q5AB87	Ribosomal 60S subunit protein L16A
201.5	A0A1D8PPS1	Ribosomal 60S subunit protein L25
187.2	Q5A0Z9	Pyruvate dehydrogenase E1 component subunit alpha (EC 1.2.4.1)
184.3	Q5AMT7	Bfr1p
177.0	A0A1D8PDZ1	Ribosomal 60S subunit protein L34B
155.7	Q5A0V9	rRNA methyltransferase
151.9	A0A1D8PF79	Glutamate decarboxylase (EC 4.1.1.15)
147.9	A0A1D8PN83	Ribosomal 40S subunit protein S11A
133.9	A0A1D8PDT4	Ribosomal 60S subunit protein L39
126.4	A0A1D8PCQ5	Ribosomal 60S subunit protein L26B
115.5	A0A1D8PRR7	Acetyl-CoA carboxylase
109.8	Q59TE0	Ribosomal 60S subunit protein L17B
106.9	Q59LS1	Ribosomal 60S subunit protein L3
105.3	Q5AML3	Oxidoreductase
103.3	O59931	60S ribosomal protein L13
98.4	A0A1D8PHH4	Ribosomal 60S subunit protein L33A
81.6	Q5AK53	ATP-dependent 6-phosphofructokinase (ATP-PFK) (Phosphofructokinase) (EC 2.7.1.11) (Phosphohexokinase)
80.7	Q5AGX8	Acetyltransferase component of pyruvate dehydrogenase complex (EC 2.3.1.12)
75.6	A0A1D8PLC9	60S ribosomal protein L20
71.5	A0A1D8PMV9	Threonine--tRNA ligase
70.1	A0A1D8PNQ6	Ribosomal 40S subunit protein S25B
68.3	O43101	Centromere/microtubule-binding protein CBF5 (Centromere-binding factor 5) (H/ACA snoRNP protein CBF5) (Small nucleolar RNP protein CBF5)
68.2	A0A1D8PMH8	Glutamate dehydrogenase
67.6	A0A1D8PR93	Histone
67.5	A0A1D8PF56	H/ACA ribonucleoprotein complex subunit

65.6	Q59S06	Nucleolar protein 58
63.2	Q5A6R1	Ribosomal protein L15
62.7	Q5AKV6	Pdx1p
62.6	A0A1D8PF08	Ribosomal 60S subunit protein L2A
62.4	A0A1D8PH21	60S ribosomal protein L36
58.0	A0A1D8PI15	Ribosomal 40S subunit protein S10A
57.4	A0A1D8PPE0	Ribosomal 40S subunit protein S13
56.7	A0A1D8PCX8	60S ribosomal protein L6
56.2	Q5A782	Translation initiation factor eIF5B
56.1	Q59PR9	Transcriptional regulator HMO1 (High mobility group protein 1)
54.0	A0A1D8PK43	Ribosomal 60S subunit protein L18A
53.6	Q5AMQ5	Carnitine O-acetyltransferase
53.3	Q59VN2	Histone H3.1/H3.2
51.3	Q59VN4	Histone H4
48.1	Q5A5V6	Pyruvate dehydrogenase E1 component subunit beta (EC 1.2.4.1)
47.9	Q5A4E2	ATP-dependent RNA helicase DED1 (EC 3.6.4.13)
47.2	Q9Y872	Sulfate adenylyltransferase (EC 2.7.7.4) (ATP-sulfurylase) (Sulfate adenylate transferase) (SAT)
45.2	A0A1D8PF11	Rpl82p
44.9	A0A1D8PK40	Ribosomal protein L19
44.0	A0A1D8PM75	Ribosomal 60S subunit protein L30
43.6	Q59QB8	Gcf1p
43.3	Q59T44	40S ribosomal protein S8
41.6	Q5ANA1	Ribosomal 60S subunit protein L8B
40.5	A0A1D8PCQ3	Eukaryotic translation initiation factor 2A (eIF-2A)
40.4	Q5A940	Multiprotein-bridging factor 1
40.2	A0A1D8PFL9	Ribosomal 60S subunit protein L14B
40.2	Q5AI86	Eukaryotic translation initiation factor 3 subunit I (eIF3i) (Eukaryotic translation initiation factor 3 39 kDa subunit homolog) (eIF-3 39 kDa subunit homolog)
40.0	Q5A5P8	Tif11p
39.1	A0A1D8PK11	RNA-binding protein
39.1	A0A1D8PCW6	Ribosomal 40S subunit protein S16A
38.9	Q5AIB8	Ribosomal 60S subunit protein L10
38.6	A0A1D8PTZ1	Uncharacterized protein
38.2	A0A1D8PGY0	Ribosomal 60S subunit protein L21A
37.8	Q59KI0	UTP--glucose-1-phosphate uridylyltransferase (EC 2.7.7.9) (UDP-glucose pyrophosphorylase) (UDPGP) (UGPase)
37.0	Q5ACM9	Eukaryotic translation initiation factor 3 subunit J (eIF3j) (Eukaryotic translation initiation factor 3 30 kDa subunit) (eIF-3 30 kDa)
36.4	A0A1D8PP59	Ubiquinol--cytochrome-c reductase subunit
36.1	Q5ALV6	40S ribosomal protein S26
35.7	A0A1D8PHG1	Oxysterol-binding protein
35.2	A0A1D8PFG4	60S ribosomal protein L27

35.2	Q59LU0	ATP-dependent RNA helicase DBP2 (EC 3.6.4.13)
35.1	Q5AGZ8	ATP-dependent 6-phosphofructokinase (ATP-PFK) (Phosphofructokinase) (EC 2.7.1.11) (Phosphohexokinase)
34.9	Q9UVJ4	60S ribosomal protein L10a
34.9	A0A1D8PPN6	Ribosomal 60S subunit protein L32
33.9	Q5AGV4	Eukaryotic translation initiation factor 3 subunit B (eIF3b) (Eukaryotic translation initiation factor 3 90 kDa subunit homolog) (eIF3 p90) (Translation initiation factor eIF3 p90 subunit homolog)
33.5	Q5AJD9	Acyl-coenzyme A oxidase
32.5	A0A1D8PP14	Ribosomal 60S subunit protein L43A
32.2	Q5AI20	Fe-S cluster-binding ribosome biosynthesis protein
32.0	A0A1D8PTR4	Ribosomal 40S subunit protein S29A
31.7	A0A1D8PU46	snoRNP complex protein
30.9	Q59TB4	Uncharacterized protein
30.8	Q96W54	40S ribosomal protein S22-A
30.5	Q5A900	Ribosomal 40S subunit protein S2
29.7	Q59ZE0	F1F0 ATP synthase subunit 4
29.5	A0A1D8PSZ0	Ife2p
29.4	Q59XW4	Acetyl-coenzyme A synthetase (EC 6.2.1.1)
29.3	A0A1D8PJ13	Bifunctional hydroxyacyl-CoA dehydrogenase/enoyl-CoA hydratase
29.3	Q5AME2	Pentafunctional AROM polypeptide [Includes: 3-dehydroquinate synthase (DHQS) (EC 4.2.3.4); 3-phosphoshikimate 1-carboxyvinyltransferase (EC 2.5.1.19) (5-enolpyruvylshikimate-3-phosphate synthase) (EPSP synthase) (EPSPS); Shikimate kinase (SK) (EC 2.7.1.71); 3-dehydroquinate dehydratase (3-dehydroquinase) (EC 4.2.1.10); Shikimate dehydrogenase (EC 1.1.1.25)]
28.5	A0A1D8PFV4	mRNA-binding protein
28.3	A0A1D8PHW1	Ribosomal 60S subunit protein L11B
27.8	Q59VR3	FK506-binding protein 3 (EC 5.2.1.8) (Peptidyl-prolyl cis-trans isomerase) (PPIase) (Rotamase)
27.5	Q59ZV5	Eukaryotic translation initiation factor 3 subunit G (eIF3g) (Eukaryotic translation initiation factor 3 RNA-binding subunit) (eIF-3 RNA-binding subunit) (Translation initiation factor eIF3 p33 subunit homolog) (eIF3 p33 homolog)
26.4	Q59PV8	ATP synthase subunit d, mitochondrial
26.4	Q5AML1	Eukaryotic translation initiation factor 3 subunit C (eIF3c) (Eukaryotic translation initiation factor 3 93 kDa subunit homolog) (eIF3 p93) (Translation initiation factor eIF3, p93 subunit homolog)
26.1	A0A1D8PRG0	Mitochondrial 54S ribosomal protein YmL2
25.6	Q5A389	Ribosomal 40S subunit protein S20
24.6	A0A1D8PSK2	40S ribosomal protein S30
24.4	A0A1D8PSV5	Ribosomal 40S subunit protein S3
24.4	A0A1D8PDL6	Ribosomal 60S subunit protein L7A
23.3	A0A1D8PGY8	Ribosomal 40S subunit protein S9B

22.7	Q5A7K0	40S ribosomal protein S24
22.5	A0A1D8PIF0	Uncharacterized protein
22.4	Q5A5Q8	40S ribosomal protein S4
22.2	A0A1D8PD83	Septin
22.0	A0A1D8PD02	Uncharacterized protein
21.2	A0A1D8PEV4	Ribosomal 60S subunit protein L42A
21.0	A0A1D8PSQ3	Type I HSP40 co-chaperone
20.8	A0A1D8PK30	Ribosomal 60S subunit protein L35A
20.6	Q5AI37	Probable metalloprotease ARX1 (EC 3.-.-.-) (Associated with ribosomal export complex protein 1)
20.6	Q5AJF7	Ribosomal 60S subunit protein L12A
20.6	A0A1D8PTH3	Alpha-ketoglutarate dehydrogenase
20.5	A0A1D8PIP0	Slr1p
20.1	Q5A7P7	F1F0 ATP synthase subunit 5
20.0	A0A1D8PEX3	DNA-directed RNA polymerase subunit (EC 2.7.7.6)
20.0	Q59YH4	Chaperonin-containing T-complex subunit
19.8	A0A1D8PL99	40S ribosomal protein S6
19.7	Q5A8Y5	DNA-directed RNA polymerase subunit beta (EC 2.7.7.6)
19.5	Q59LF9	Methionine aminopeptidase 2 (MAP 2) (MetAP 2) (EC 3.4.11.18) (Peptidase M)
19.4	A0A1D8PF45	Ribosomal protein L37
19.3	A0A1D8PE45	Nicotinate-nucleotide pyrophosphorylase [carboxylating] (EC 2.4.2.19) (Quinolinate phosphoribosyltransferase [decarboxylating])
19.0	A0A1D8PK22	Ribosomal 40S subunit protein S15
18.7	A0A1D8PKZ3	ATP-dependent RNA helicase
18.6	Q5ALV9	Cytochrome c oxidase subunit 6A, mitochondrial (Cytochrome c oxidase polypeptide VIa)
18.6	A0A1D8PQS0	60S acidic ribosomal protein P0
18.6	Q59P08	Glyoxylate reductase
18.5	Q59LZ9	Tom22p
18.5	A0A1D8PJF9	Acetolactate synthase (EC 2.2.1.6)
18.3	A0A1D8PHA3	Ubiquinol--cytochrome-c reductase catalytic subunit
18.3	Q59N40	Aspartate aminotransferase (EC 2.6.1.1)
18.0	Q5AJD0	ATP-dependent RNA helicase DBP5 (EC 3.6.4.13)
17.7	Q5AAU3	Protein transport protein SEC31
17.6	Q59U72	Ferrochelatase (EC 4.99.1.1)
17.4	A0A1D8PQQ5	Ribosomal 40S subunit protein S18B
17.4	Q9HFQ7	60S acidic ribosomal protein P1-A (CaRP1A)
17.3	Q59YC4	Chaperonin-containing T-complex subunit
17.3	Q59PL9	Eukaryotic translation initiation factor 3 subunit A (eIF3a) (Eukaryotic translation initiation factor 3 110 kDa subunit homolog) (eIF3 p110) (Translation initiation factor eIF3, p110 subunit homolog)
17.2	A0A1D8PP21	Methionine--tRNA ligase
17.0	Q5A3P4	Tryptophan--tRNA ligase

16.6	Q5A8X7	Protein FYV4, mitochondrial
16.2	A0A1D8PHD8	DNA-directed RNA polymerase core subunit
16.1	A0A1D8PQM4	DNA-directed RNA polymerase subunit beta (EC 2.7.7.6)
15.7	Q59MA9	Clustered mitochondria protein homolog (Protein TIF31 homolog)
15.6	A0A1D8PGM4	U3 small nucleolar RNA-associated protein 22
15.4	A0A1D8PCC8	Rrs1p
15.4	Q5AK00	Uncharacterized protein
15.3	A0A1D8PFV1	Ribosomal 60S subunit protein L4B
15.3	A0A1D8PDU3	Ribosomal 40S subunit protein S23B
15.3	Q5AHD3	Mitochondrial 54S ribosomal protein YmL35
15.1	A0A1D8PSI3	Uncharacterized protein
15.1	A0A1D8PQM1	Single-stranded telomeric DNA-binding/mRNA-binding protein
14.8	A0A1D8PCY5	Septin
14.4	A0A1D8PDK6	6,7-dimethyl-8-ribityllumazine synthase (DMRL synthase) (EC 2.5.1.78)
14.2	A0A1D8PEZ0	Asr1p
14.2	A0A1D8PTK1	Chorismate synthase (EC 4.2.3.5)
14.0	P39827	Cell division control protein 10
14.0	Q5APD2	Malate synthase (EC 2.3.3.9)
13.9	Q5A6A1	Ribosomal 60S subunit protein L24A
13.6	A0A1D8PTM0	Uncharacterized protein
13.3	A0A1D8PRK7	Uncharacterized protein
13.0	Q5AGZ7	Ribosomal 60S subunit protein L5
12.9	A0A1D8PSB9	Srp40p
12.9	A0A1D8PLA3	rRNA-processing protein
12.9	P40910	40S ribosomal protein S1 (S3aE)
12.8	A0A1D8PR11	Uncharacterized protein
12.7	A0A1D8PU51	Uncharacterized protein
12.6	Q5A1D5	FACT complex subunit SPT16 (CaCDC68) (Cell division control protein 68) (Facilitates chromatin transcription complex subunit SPT16)
12.4	A0A1D8PET7	Uncharacterized protein
12.3	A0A1D8PUA6	DNA-directed RNA polymerase subunit (EC 2.7.7.6)
12.2	Q59YH5	Mitochondrial 54S ribosomal protein YmL36
12.2	A0A1D8PJF3	Uncharacterized protein
12.1	P53704	Glutamine--fructose-6-phosphate aminotransferase [isomerizing] (GFAT) (EC 2.6.1.16) (D-fructose-6-phosphate amidotransferase) (Hexosephosphate aminotransferase)
12.0	A0A1D8PCH2	mRNA-binding protein
11.8	Q5A6S0	Mitochondrial 54S ribosomal protein YmL40
11.7	A0A1D8PMD5	Uncharacterized protein
11.6	Q59R18	Asparagine--tRNA ligase
11.5	Q5AKX2	Fumarate reductase
11.5	Q5A1Z1	Mitochondrial 37S ribosomal protein MRP21
11.4	Q5AEN2	Ribosomal 60S subunit protein L9B

11.3	Q59M32	Mitochondrial 54S ribosomal protein YmL15
11.3	Q5A678	Ssz1p
11.3	A0A1D8PKK1	Translocon subunit
11.2	Q5AF38	Alpha-mannosidase (EC 3.2.1.-)
11.2	A0A1D8PDE3	Coatomer subunit gamma
11.2	Q59NG6	Mitochondrial 37S ribosomal protein PET123
11.2	Q5A0W7	RuvB-like helicase 1 (EC 3.6.4.12)
10.9	A0A1D8PRJ6	Translation initiation factor eIF2 subunit beta
10.9	Q5ADQ6	40S ribosomal protein S12
10.8	Q5AAS9	Mitochondrial 54S ribosomal protein YmL19
10.8	Q5AQ76	Protein transport protein SEC24
10.7	Q9B8D8	Cytochrome c oxidase subunit 2 (EC 1.9.3.1) (Cytochrome c oxidase polypeptide II)
10.6	A0A1D8PSC5	Ribosomal 60S subunit protein L28
10.6	Q5AJ93	40S ribosomal protein S7
10.3	Q59ZE2	2-methoxy-6-polyprenyl-1,4-benzoquinol methylase, mitochondrial (EC 2.1.1.201) (Ubiquinone biosynthesis methyltransferase COQ5)
10.1	Q5AGZ9	RuvB-like helicase 2 (EC 3.6.4.12)
9.9	A0A1D8PQH1	Uncharacterized protein
9.8	Q5AK02	Mitochondrial 37S ribosomal protein RSM7
9.8	Q5AF98	Zuotin
9.8	A0A1D8PPT5	Ribosomal 60S subunit protein L23B
9.7	A0A1D8PHF5	Ribosomal 60S subunit protein L31B
9.7	A0A1D8PND4	Mitochondrial 37S ribosomal protein YMR31
9.7	A0A1D8PMK0	Ago1p
9.6	Q5A779	GTP-binding protein
9.6	A0A1D8PDT3	Ribosomal 40S subunit protein S14B
9.6	Q5A3N5	Mitochondrial 54S ribosomal protein YmL13
9.4	Q59YE8	Translation termination factor GTPase eRF3
9.3	Q5ADU3	Eukaryotic translation initiation factor 3 subunit H (eIF3h)
9.2	O74161	Chitin biosynthesis protein CHS5
9.1	Q5AK16	T-complex protein 1 subunit gamma
9.1	Q5A798	Mitochondrial 54S ribosomal protein YmL3
9.1	A0A1D8PJB0	Coatomer subunit alpha
9.1	A0A1D8PNC8	Proteasome subunit beta (EC 3.4.25.1)
9.1	Q59UQ4	Chromatin-binding transcription coactivator
9.0	A0A1D8PHP8	Putative ammonium permease
9.0	A0A1D8PRF5	Sla2p
9.0	A0A1D8PJA6	Uncharacterized protein
8.9	A0A1D8PTP9	Chaperone ATPase
8.9	Q5AEF2	Protein transport protein SEC13
8.7	Q5A1E3	Transcriptional regulator CBF1
8.6	A0A1D8PTY4	Elf1p
8.5	Q5AK42	rRNA-processing protein EFG1

8.5	Q59ZH8	Translation termination factor eRF1
8.4	Q5A455	Protein transport protein SEC23
8.4	Q5ALL8	FACT complex subunit POB3 (Facilitates chromatin transcription complex subunit POB3)
8.4	Q5ANB2	ATP-dependent RNA helicase DBP10 (EC 3.6.4.13)
8.4	Q59VP1	Histone H2B.2
8.4	A0A1D8PMN9	Chaperonin-containing T-complex subunit
8.3	Q59ZI7	Mitochondrial 54S ribosomal protein YmL10/YmL18
8.2	Q5A6M9	SnoRNA-binding protein
8.2	A0A1D8PR73	Uncharacterized protein
8.1	Q5AP79	Mir1p
8.1	Q5A2T2	ATP-binding cassette family ATPase
8.1	O94150	37S ribosomal protein S9, mitochondrial
8.0	P83781	Mitochondrial outer membrane protein porin (Cytoplasmic antigenic protein 4)
8.0	Q5A516	ADP/ATP carrier protein
7.9	A0A1D8PN45	Dynamin-like GTPase
7.8	A0A1D8PG09	Stf2p
7.7	A0A1D8PPV5	Coatomer subunit beta'
7.7	A0A1D8PH91	Mitochondrial 37S ribosomal protein MRP51
7.5	Q5AK79	CTP synthase (EC 6.3.4.2) (UTP--ammonia ligase)
7.5	Q5ALN3	Uncharacterized protein
7.4	Q5A4Y4	Uncharacterized protein
7.4	Q59X93	Mitochondrial 54S ribosomal protein YmL17/YmL30
7.4	A0A1D8PK61	Ribosomal 40S subunit protein S19A
7.3	Q59P03	NADH-cytochrome b5 reductase 1 (EC 1.6.2.2) (Microsomal cytochrome b reductase)
7.3	A0A1D8PP33	Uncharacterized protein
7.3	A0A1D8PQC8	Uncharacterized protein
7.2	Q59QD6	Elongation factor 1-alpha 2 (EF-1-alpha 2)
7.1	Q5ABD0	Vacuolar-sorting protein SNF7 (Vacuolar protein-sorting-associated protein 32)
7.1	Q5A4L1	Uncharacterized protein
7.0	Q5AJU7	AP-1-like transcription factor CAP1
7.0	A0A1D8PSW1	Pex14p
7.0	Q5A7M1	ADP-ribosylation factor GTPase-activating protein
7.0	A0A1D8PGA2	Protein phosphatase regulator
6.9	Q59MZ8	DNA-directed RNA polymerase core subunit
6.9	Q5ACU9	Transcription factor
6.8	Q5A0M4	Elongation factor 2 (EF-2)
6.7	A0A1D8PK85	Proteasome core particle subunit beta 1
6.7	Q5A4L3	Uncharacterized protein
6.7	Q5A7K7	Uncharacterized protein
6.7	Q59LZ5	Protein channel
6.7	A0A1D8PJ76	Uncharacterized protein

6.6	Q59YF4	Increased recombination centers protein 22-1
6.6	Q59QN7	Succinate dehydrogenase [ubiquinone] iron-sulfur subunit, mitochondrial (EC 1.3.5.1)
6.6	A0A1D8PJ20	Proteasome endopeptidase complex (EC 3.4.25.1)
6.6	A0A1D8PEY9	Ribosomal 40S subunit protein S17B
6.5	A0A1D8PH72	Uncharacterized protein
6.5	A0A1D8PFZ9	U3 small nucleolar ribonucleoprotein protein MPP10
6.4	Q5AA47	Arp2/3 complex 34 kDa subunit
6.4	A0A1D8PGR5	Uncharacterized protein
6.4	A0A1D8PRM5	F1F0 ATP synthase subunit f
6.4	A0A1D8PL86	Mlp1p
6.3	Q5A6R2	Bifunctional phosphoribosylaminoimidazolecarboxamide formyltransferase/IMP cyclohydrolase
6.3	Q59Y36	Uncharacterized protein
6.3	P12461	Thymidylate synthase (TS) (TSase) (EC 2.1.1.45)
6.3	Q5AEI1	Nuo2p
6.2	Q5AI14	Trehalose-phosphatase
6.2	Q59RP7	54S ribosomal protein L4, mitochondrial
6.2	Q5AFG1	Ribosome biogenesis protein ALB1
6.1	Q5AHA6	AMP deaminase
6.1	A0A1D8PQJ8	Uncharacterized protein
6.1	A0A1D8PRA6	Uncharacterized protein
6.1	Q5ABS1	Cytochrome b-c1 complex subunit 7
6.0	Q59Z11	Actin-related protein 3
6.0	A0A1D8PQN0	Ribosomal 40S subunit protein S28B
6.0	Q5ABZ2	Transcription factor RBF1 (RPG-box-binding factor 1)
5.9	Q59Z65	Proteasome core particle subunit beta 5
5.9	Q9UVL1	Non-histone chromosomal protein 6
5.9	Q5A5V8	Uncharacterized protein
5.8	Q5ALV5	Cytochrome c oxidase subunit IV
5.8	A0A1D8PFH2	Ribosome biosynthesis protein
5.7	Q5ANL6	13 kDa ribonucleoprotein-associated protein
5.7	A0A1D8PLR7	NADPH--cytochrome P450 reductase (CPR) (p450R) (EC 1.6.2.4)
5.6	A0A1D8PTY6	Proteasome endopeptidase complex (EC 3.4.25.1)
5.6	A0A1D8PDE2	Aldo-keto reductase superfamily protein
5.5	Q5ALL3	tRNA-dihydrouridine(47) synthase [NAD(P)(+)] (EC 1.3.1.89) (tRNA-dihydrouridine synthase 3)
5.5	Q5AAW3	ATP-dependent RNA helicase DHH1 (EC 3.6.4.13)
5.4	A0A1D8PTD1	Bifunctional carbamoylphosphate synthetase/aspartate transcarbamylase
5.4	A0A1D8PNY3	Uncharacterized protein
5.4	P53698	Cytochrome c
5.4	A0A1D8PLW8	Proteasome regulatory particle lid subunit
5.4	A0A1D8PMJ1	RNA-binding GTPase
5.3	A0A1D8PCU5	Uncharacterized protein

5.3	A0A1D8PDC7	Uncharacterized protein
5.2	A0A1D8PFP3	5'-3' exoribonuclease 1 (EC 3.1.13.-)
5.1	Q874I4	Dihydroorotate dehydrogenase (quinone), mitochondrial (DHOD) (DHODase) (DHOdehase) (EC 1.3.5.2) (Dihydroorotate oxidase)
5.1	A0A1D8PMP9	Methionine aminopeptidase (EC 3.4.11.18)
5.1	Q59YJ9	mRNA-binding translational activator
5.0	A0A1D8PRA1	Mitochondrial 54S ribosomal protein YmL49
5.0	Q5AK59	ATP-dependent RNA helicase HAS1 (EC 3.6.4.13)
5.0	A0A1D8PNN8	Cam1-1p
4.9	Q59QC1	Mitochondrial 54S ribosomal protein YmL28
4.9	Q8NJN3	Acetyl-coenzyme A synthetase 2 (EC 6.2.1.1) (Acetate--CoA ligase 2) (Acyl-activating enzyme 2)
4.9	A0A1D8PU67	Proteasome core particle subunit beta 2
4.9	Q59K70	Uncharacterized protein
4.8	A0A1D8PQ03	Uncharacterized protein
4.8	Q3MNT0	Transcription elongation factor SPT6 (Chromatin elongation factor SPT6)
4.8	Q59RQ6	Dihydrolipoyl dehydrogenase (EC 1.8.1.4)
4.8	Q5A501	ESCRT-III subunit protein
4.7	Q59SM8	Trifunctional formate-tetrahydrofolate ligase/methenyltetrahydrofolate cyclohydrolase/methylenetetrahydrofolate dehydrogenase
4.7	Q5A8Y6	Mitochondrial 54S ribosomal protein YmL23
4.7	A0A1D8PJA9	Uncharacterized protein
4.7	A0A1D8PM44	Methylenetetrahydrofolate dehydrogenase (NAD(+))
4.7	A0A1D8PPJ1	Cic1p
4.6	A0A1D8PQJ1	Uncharacterized protein
4.6	A0A1D8PEV9	Mitochondrial 37S ribosomal protein MRPS5
4.6	A0A1D8PIF1	Uncharacterized protein
4.6	A0A1D8PHT4	Uncharacterized protein
4.6	Q59N42	Serine/threonine-protein phosphatase (EC 3.1.3.16)
4.6	A0A1D8PLQ3	Gly-Xaa carboxypeptidase
4.6	A0A1D8PG96	Hsp70 family ATPase
4.6	Q5A8Z9	Uncharacterized protein
4.5	A0A1D8PND9	Coatomer subunit delta
4.5	Q59XP0	Protein transport protein SEC9
4.5	Q92206	Squalene monooxygenase (EC 1.14.14.17) (Squalene epoxidase) (SE)
4.5	Q5A6P2	RNA cytidine acetyltransferase (EC 2.3.1.-) (18S rRNA cytosine acetyltransferase)
4.4	A0A1D8PKD3	Nucleolar GTP-binding protein 1
4.4	A0A1D8PPQ1	Tif3p
4.4	A0A1D8PHY2	Mitochondrial 54S ribosomal protein YmL7/YmL5
4.4	Q5AG43	Ribosomal 40S subunit protein S5
4.4	Q92410	Alpha,alpha-trehalose-phosphate synthase [UDP-forming] (EC

		2.4.1.15) (Trehalose-6-phosphate synthase) (UDP-glucose-glucosephosphate glucosyltransferase)
4.3	A0A1D8PRL4	Mis12p
4.3	Q5APB9	Uncharacterized protein
4.3	Q59Y40	SnoRNA-binding rRNA-processing protein
4.3	Q5A222	NADH-ubiquinone oxidoreductase
4.3	Q5A397	Hsp70 family ATPase
4.3	Q5A302	Endoplasmic reticulum vesicle protein 25
4.3	Q5A3P1	Exosome catalytic subunit
4.2	A0A1D8PRU4	Actin-related protein 2/3 complex subunit 4
4.2	A0A1D8PI73	Translation initiation factor eIF4G
4.2	Q5A4X9	Ribonuclease
4.2	A0A1D8PHI0	Replication factor C subunit 1
4.2	A0A1D8PF50	18S rRNA pseudouridine methyltransferase
4.1	A0A1D8PE97	Glycine cleavage system P protein (EC 1.4.4.2)
4.1	A0A1D8PSN8	rRNA (Cytosine-C5-)-methyltransferase
4.1	A0A1D8PJ73	Ali1p
4.1	Q5AJA5	DEAH-box ATP-dependent RNA helicase
4.1	A0A1D8PFJ8	DNA-directed RNA polymerase core subunit
4.1	A0A1D8PK71	Uncharacterized protein
4.1	Q59LF3	Regulator of cytoskeleton and endocytosis RVS167
4.0	Q59KZ3	Uridylate kinase (UK) (EC 2.7.4.14) (ATP:UMP phosphotransferase) (Deoxycytidylate kinase) (CK) (dCMP kinase) (Uridine monophosphate kinase) (UMP kinase) (UMPK)
4.0	Q59PZ1	Proteasome endopeptidase complex (EC 3.4.25.1)
4.0	Q5AI30	Mitochondrial 37S ribosomal protein RSM24
4.0	Q5AH14	Tom40p
4.0	P10613	Lanosterol 14-alpha demethylase (EC 1.14.13.70) (CYPLI) (Cytochrome P450 51) (Cytochrome P450-14DM) (Cytochrome P450-LIA1) (Sterol 14-alpha demethylase)
4.0	A0A1D8PT60	Arc40p
3.9	A0A1D8PCP4	Uncharacterized protein
3.9	A0A1D8PU27	Uncharacterized protein
3.9	A0A1D8PQQ7	mRNA-binding protein
3.9	A0A1D8PG16	Ribosomal 60S subunit protein L38
3.9	Q5ADP3	Transcription regulator
3.9	Q5A1E8	Succinate dehydrogenase [ubiquinone] flavoprotein subunit, mitochondrial (EC 1.3.5.1)
3.9	Q5AEE1	Histone H2A.Z
3.9	A0A1D8PIS4	Trehalose 6-phosphate synthase/phosphatase complex subunit
3.9	Q5AK62	Virulence protein SSD1
3.8	A0A1D8PMA9	Uncharacterized protein
3.8	Q5ADR2	Glutamate-5-semialdehyde dehydrogenase
3.8	Q5ALK3	Ribose phosphate diphosphokinase subunit
3.8	Q59QB7	Chaperonin-containing T-complex alpha subunit
3.8	Q59KY7	Mitochondrial 37S ribosomal protein RSM18

3.7	Q5A9A9	RNA-binding signal recognition particle subunit
3.7	A0A1D8PUB4	L-iditol 2-dehydrogenase
3.7	Q59RN2	Actin-related protein 2/3 complex subunit 5
3.7	A0A1D8PRR4	Trans-hexaprenyltranstransferase
3.7	A0A1D8PEA2	Dolichyl-phosphate beta-D-mannosyltransferase
3.7	A0A1D8PGL1	Uncharacterized protein
3.7	A0A1D8PHC4	Uncharacterized protein
3.7	Q5A0N3	Ribonucleoside-diphosphate reductase (EC 1.17.4.1)
3.6	A0A1D8PR80	Ran guanyl-nucleotide exchange factor
3.6	Q5APD0	Uncharacterized protein
3.5	A0A1D8PEY6	Tricalbin
3.5	A0A1D8PQE5	RNA export factor
3.5	A0A1D8PHE0	Mitochondrial 37S ribosomal protein MRPS35
3.5	Q5A8H8	Pbp2p
3.5	Q5AFE4	Regulator of cytoskeleton and endocytosis RVS161
3.5	A0A1D8PMK4	Uncharacterized protein
3.5	Q5AL45	Elongation factor G, mitochondrial (EF-Gmt) (Elongation factor G 1, mitochondrial) (mEF-G 1) (Elongation factor G1)
3.5	A0A1D8PH93	Uncharacterized protein
3.5	A0A1D8PMT6	Coatomer subunit epsilon
3.5	A0A1D8PCC3	Transcriptional regulator
3.5	A0A1D8PNA3	Peptide alpha-N-acetyltransferase complex A subunit
3.5	A0A1D8PJX3	Cytochrome b-c1 complex subunit Rieske, mitochondrial (EC 1.10.2.2)
3.4	A0A1D8PE03	Uncharacterized protein
3.4	Q59UF7	Aspartate--tRNA ligase
3.4	Q5AH35	Mitochondrial 54S ribosomal protein MRP49
3.4	Q59WV9	Uncharacterized protein
3.4	A0A1D8PIR2	Gcn1p
3.3	A0A1D8PE79	Oxysterol-binding protein related protein
3.3	A0A1D8PDD1	Lipid-binding protein
3.3	Q5ABC3	Elongation factor Tu
3.3	A0A1D8PLU5	Csh3p
3.2	A0A1D8PG50	F1F0 ATP synthase subunit i
3.2	A0A1D8PQQ9	Mitochondrial 54S ribosomal protein YmL6
3.2	O94083	Eukaryotic translation initiation factor 5A (eIF-5A) (eIF-4D)
3.2	Q5A006	Coatomer subunit zeta
3.2	Q5AAR2	Uncharacterized protein
3.2	Q8TGH6	Guanosine-diphosphatase (GDPase) (EC 3.6.1.42)
3.1	A0A1D8PMP1	Glycerol-3-phosphate dehydrogenase (EC 1.1.5.3)
3.1	Q59T87	Uncharacterized protein
3.1	A0A1D8PFX2	DNA-directed RNA polymerase core subunit
3.1	Q5A8X6	Succinate--CoA ligase [ADP-forming] subunit alpha, mitochondrial (EC 6.2.1.5) (Succinyl-CoA synthetase subunit alpha) (SCS-alpha)

3.1	A0A1D8PC73	Uncharacterized protein
3.0	A0A1D8PL02	F1F0 ATP synthase subunit e
3.0	Q5A6Q4	tRNA (adenine(58)-N(1))-methyltransferase non-catalytic subunit TRM6 (tRNA(m1A58)-methyltransferase subunit TRM6) (tRNA(m1A58)MTase subunit TRM6)
3.0	Q59KV8	Lipid-binding protein
3.0	A0A1D8PLN1	rRNA-processing protein
3.0	A0A1D8PTN4	snoRNP complex protein
3.0	Q59Z25	Mitochondrial nucleoid protein
3.0	Q5ADP0	Histone deacetylase (EC 3.5.1.98)
3.0	A0A1D8PQ55	Uncharacterized protein
3.0	A0A1D8PG26	Uncharacterized protein
3.0	A0A1D8PRH3	Ume1p
3.0	Q5AF71	Putative phosphotransferase
2.9	Q59VX8	Septation protein 7 (Seventh homolog of septin 1)
2.9	A0A1D8PM35	Translation elongation factor 1 subunit beta
2.9	Q5ADT9	37S ribosomal protein S10, mitochondrial (Mitochondrial ribosomal small subunit protein 10)
2.9	Q59TD5	NADH dehydrogenase [ubiquinone] flavoprotein 1, mitochondrial (EC 1.6.5.3) (EC 1.6.99.3)
2.9	A0A1D8PHH2	Png2p
2.9	A0A1D8PSC8	Arc1p
2.8	Q59RB8	Isocitrate lyase
2.8	P25997	Elongation factor 3 (EF-3)
2.8	A0A1D8PLK1	Proteasome core particle subunit beta 6
2.8	A0A1D8PPW9	Aminomethyltransferase (EC 2.1.2.10) (Glycine cleavage system T protein)
2.8	Q5APK5	Cytochrome c oxidase subunit Va
2.8	A0A1D8PTX1	Actin-related protein 2
2.8	A0A1D8PND7	Uncharacterized protein
2.8	Q59RK3	Putative serine--tRNA ligase
2.7	Q5ANH5	Ribosomal protein P2B
2.7	A0A1D8PU56	Long-chain fatty acid-CoA ligase
2.7	Q5AJZ5	Proteasome core particle subunit beta 4
2.7	A0A1D8PQL8	Glutamate--tRNA ligase
2.7	Q5A6S7	Uncharacterized protein
2.7	A0A1D8PRH6	Proteasome endopeptidase complex (EC 3.4.25.1)
2.7	Q5A5S6	Malate dehydrogenase (EC 1.1.1.37)
2.7	A0A1D8PSE0	Uncharacterized protein
2.6	Q5A7P6	Mitochondrial 54S ribosomal protein RML2
2.6	Q5A2A2	Mitochondrial homologous recombination protein 1
2.6	Q59XV1	Calmodulin-dependent protein kinase
2.6	Q5AAI8	Nucleosome assembly protein 1
2.6	P83782	Cytochrome b-c1 complex subunit 2, mitochondrial (Complex III subunit 2) (Core protein II) (Cytoplasmic antigenic protein 5) (Ubiquinol-cytochrome-c reductase complex core protein 2)

2.6	Q59Z24	DNA-directed RNA polymerase subunit
2.6	A0A1D8PTI9	Rgd3p
2.6	Q5ANP6	Sbp1p
2.6	Q59X67	Enhanced filamentous growth protein 1
2.6	A0A1D8PJK2	D-aminoacyl-tRNA deacylase (EC 3.1.1.-) (EC 3.1.1.96)
2.6	A0A1D8PQD5	Cytochrome c oxidase subunit
2.5	A0A1D8PE54	Bbc1p
2.5	A0A1D8PKC3	Translation elongation factor EF1B gamma
2.5	O42817	40S ribosomal protein S0
2.5	Q5ADU2	Lysine--tRNA ligase (EC 6.1.1.6) (Lysyl-tRNA synthetase)
2.5	O93827	Mannose-1-phosphate guanyltransferase (EC 2.7.7.13) (ATP-mannose-1-phosphate guanylyltransferase) (CASRB1) (GDP-mannose pyrophosphorylase)
2.5	Q5A4Q1	Adenylate kinase (EC 2.7.4.3) (ATP-AMP transphosphorylase) (ATP:AMP phosphotransferase) (Adenylate kinase cytosolic and mitochondrial) (Adenylate monophosphate kinase)
2.4	A0A1D8PTS0	Ribosomal protein P2A
2.4	Q59M49	mRNA-binding ribosome synthesis protein
2.4	Q5A473	Gvp36p
2.3	Q59XU9	Glycerol-3-phosphate dehydrogenase [NAD(+)] (EC 1.1.1.8)
2.3	A0A1D8PQ38	Uncharacterized protein
2.3	A0A1D8PD15	Proteasome regulatory particle lid subunit
2.3	A0A1D8PD99	mRNA-binding ribosome biosynthesis protein
2.3	Q59QT0	DNA-directed RNA polymerase core subunit
2.2	Q5AMR4	Exosome non-catalytic core subunit
2.2	Q59SI1	ATPase-activating ribosome biosynthesis protein
2.2	A0A1D8PFS0	SNAP receptor
2.1	Q00310	Glycolipid 2-alpha-mannosyltransferase 1 (EC 2.4.1.-) (Alpha-1,2-mannosyltransferase 1)
2.1	A0A1D8PDC4	ATP synthase subunit alpha
2.1	A0A1D8PQP7	Cyb5p
2.1	Q5A850	Glycogen [starch] synthase (EC 2.4.1.11)
2.1	A0A1D8PGU0	Hsp70 family chaperone
2.0	A0A1D8PTI7	40S ribosomal protein S27
2.0	O93852	D-arabinono-1,4-lactone oxidase (ALO) (EC 1.1.3.37) (L-galactono-gamma-lactone oxidase)
2.0	A0A1D8PTG5	U4/U6-U5 snRNP complex subunit
2.0	G1UB61	Septin CDC11 (Cell division control protein 11)
2.0	P0CT51	Blood-induced peptide 1
2.0	A0A1D8PL12	Uncharacterized protein
2.0	Q5A109	Ubiquitin-ribosomal 40S subunit protein S31 fusion protein
2.0	Q59M70	NADH-cytochrome b5 reductase 2 (EC 1.6.2.2) (Mitochondrial cytochrome b reductase)
2.0	A0A1D8PRY3	ATP synthase subunit gamma
2.0	A0A1D8PCT4	Phenylalanine--tRNA ligase subunit alpha
1.9	A0A1D8PDI5	Ubiquitin-binding protein

1.9	Q5AFA8	Abp1p
1.9	Q5AHY9	DNA polymerase epsilon noncatalytic subunit
1.9	A0A1D8PDE8	Obg-like ATPase 1
1.9	A0A1D8PGX7	Uncharacterized protein
1.8	A0A1D8PC43	Diphosphomevalonate decarboxylase (EC 4.1.1.33) (Mevalonate pyrophosphate decarboxylase)
1.8	Q5ACI8	Peptidyl-prolyl cis-trans isomerase D (PPIase D) (EC 5.2.1.8) (Rotamase D)
1.8	Q5AHB1	Actin cytoskeleton-regulatory complex protein PAN1
1.8	Q59Y38	Uncharacterized protein
1.8	A0A1D8PKZ9	ATP synthase subunit beta (EC 3.6.3.14)
1.7	A0A1D8PRP0	U4/U6-U5 snRNP complex subunit
1.7	A0A1D8PS79	Isocitrate dehydrogenase [NADP] (EC 1.1.1.42)
1.7	P0CY34	Transcriptional repressor TUP1
1.7	Q5A7P9	Thioredoxin peroxidase
1.7	A0A1D8PR99	Protein disulfide isomerase
1.7	A0A1D8PMH6	Tryptophan synthase (EC 4.2.1.20)
1.7	Q5A9D9	Homoisocitrate dehydrogenase
1.7	A0A1D8PJ67	Uncharacterized protein
1.7	A0A1D8PHU0	Mitochondrial 54S ribosomal protein MRPL50
1.6	Q5AMQ2	Clathrin light chain
1.6	Q5A893	F-actin-capping protein subunit alpha
1.6	Q5A3K7	Phosphoglycerate dehydrogenase
1.6	A0A1D8PKE2	Mitogen-activated protein kinase kinase
1.6	A0A1D8PPH3	Uncharacterized protein
1.6	A0A1D8PHF8	Wh11p
1.6	A0A1D8PTZ6	Uncharacterized protein
1.6	Q5ADT0	Slk19p
1.6	Q59NP1	Copper transport protein CTR1
1.6	Q59TU0	Nascent polypeptide-associated complex subunit beta (NAC-beta) (Beta-NAC)
1.6	A0A1D8PKD0	Uncharacterized protein
1.5	A0A1D8PG81	Hgt7p
1.5	O13426	Serine hydroxymethyltransferase, cytosolic (SHMT) (EC 2.1.2.1) (Glycine hydroxymethyltransferase) (SHMII) (Serine methylase)
1.5	P83774	Guanine nucleotide-binding protein subunit beta-like protein (Cytoplasmic antigenic protein 1)
